# Social vulnerability and factors associated with oral impact on daily performance among adolescents

**DOI:** 10.1186/s12955-017-0746-1

**Published:** 2017-08-30

**Authors:** Inara Pereira da Cunha, Antônio Carlos Pereira, Antônio Carlos Frias, Vladen Vieira, Marcelo de Castro Meneghim, Marília Jesus Batista, Karine Laura Cortellazzi, Jaqueline Vilela Bulgareli

**Affiliations:** 1 Department of Community Dentistry, School of Dentistry of Piracicaba, State University of Campinas, Av. Limeira, 901, P.O. BOX 52, Piracicaba, SP 13414-903 Brazil; 2 Department of Public Health, Jundiaí Medical School, São Paulo, Brazil, Rua Francisco Telles, 255, P.O. BOX 1295, Jundiaí, SP 13202-550 Brazil; 3 Faculty of Dentistry, University of São Paulo, São Paulo, Brazil, Av. Prof. Lineu Prestes 2227 Butantã 05508-900, Sao Paulo, SP Brazil; 4 The state of São Paulo Secretary of Health, Sao Paulo, Brazil

**Keywords:** Oral health, Quality of life, Adolescent, Social vulnerability

## Abstract

**Background:**

Oral disorders may negatively affect the quality of life (QoL) of adolescents.

To investigate how social vulnerability and oral-health status factors affect QoL in 15–19 years olds who participated in the “SB São Paulo 2015” state survey.

**Methods:**

The relationship of several independent variables, namely Paulista Social Vulnerability Index (PSVI) score, gender, skin color, family income, age, untreated caries, tooth loss [determined by the Decayed, Missing, Filled-Teeth (DMF-T) index], toothache, periodontal condition [determined by the Community Periodontal Index (CPI)], and malocclusion (maxillary overjet, cross bite, or open bite) affect daily life, measured by the Oral Impacts on Daily Performance (OIDP) instrument. Logistic regression analyses were carried out based on a hierarchical model.

**Results:**

The final sample consisted of 5402 adolescents. The prevalence of at least one negative impact of oral health on QoL was 37.3%. After adjustment, demographic factors that were found to influence this impact significantly (*p* < 0.01) were female gender [odds ratio (OR) 1.78, 95% confidence interval (CI) = 1.59–2.0], non-white skin color (OR 1.66, 95% CI = 1.47–1.88), and a low family income (OR 1.28, 95% CI = 1.28–1.29). Additionally, oral conditions associated with oral health impact on QoL included the presence of at least one untreated tooth decay lesion (OR 1.42, 95% CI = 1.25–1.61), loss of at least one tooth (OR 1.49; 95% CI = 1.25–1.78), toothache (OR 4.87, 95% CI = 4.25–5.59), bleeding on probing (OR 1.45, 95% CI = 1.25–1.68), and severe maxillary overjet (OR 1.68, 95% CI = 1.15–2.45).

**Conclusion:**

Social vulnerability (PSVI score) was not associated with the OIDP score, but oral health conditions and socio-demographic variables, including gender, skin color, and income, were found to affect adolescents’ daily activities. Strategies that consider the perceptions of this segment of the population should be implemented to strengthen their autonomy and totality of care.

## Background

With the recent trend toward individualization of medicine, efforts to consider age in treatment planning have been increasing. Providing health care for teenagers is particularly challenging given the extensive transformation and maturation that occurs during adolescence [1]. Because adolescents are at increased risk for some oral diseases, it is necessary that quality dental care services for this population aimed at improving oral-health behavior be developed together with communities [2]. Valid indicators from a situational analysis of real-world conditions must be developed for this purpose [3].

Recent national epidemiological surveys in Brazil have revealed an interesting issue with respect to oral diseases in childhood and adolescence [4, 5]. Notably, in 2010, 12-year-old Brazilian children were found to have, on average, 2.07 teeth with dental caries (active or filled), whereas 15 to 19-year-old adolescents had, on average, 4.25 teeth with caries. Regarding periodontal changes, bleeding on probing (BoP) of the gums were identified in 11.7% of 12 year olds and in 28.4% of adolescents. The presence of shallow and deep pockets was found to be an aggravating factor for these periodontal changes. The prevalence of malocclusion was similar for the two age groups [5]. Given these trends of marked worsening of oral health over an age difference of just 3 years, it is necessary to elucidate the factors underlying these trends to avoid their perpetuation and repercussions in adulthood.

Generally, studies of oral diseases per se have not considered the impacts of oral health problems on daily activities and, by extension, quality of life (QoL). The perceptions of individual patients must be assessed to reveal attitudes about these conditions and the degree of life disruption produced by them [6]. Aimed at elucidating this issue, epidemiological surveys have investigated the perception of the impact of oral diseases on QoL with instruments such as the Oral Impacts on Daily Performance (OIDP) [7–10]. Surveys have indicated that untreated dental caries and malocclusion are the main problems affecting teenagers [11, 12]. Other factors, such as tooth loss, toothache [8], and bleeding gums have also been reported [10, 13]. The effects of these clinical changes include oral discomfort, functional limitations, impaired school performance, and socialization difficulties, issues that may compromise QoL [13, 14].

Social context, specifically situations of vulnerability, may be related to the perceived impact of oral health on daily activities. Vulnerability is a construct that considers disease responses beyond the realm of pathogenic action. Accordingly, disease exposure likelihood has been linked to individual, collective, and contextual aspects that generate greater susceptibility or greater protection, depending on the availability of resources from different domains [15].

In general, vulnerability among adolescents manifests itself as violence, a lack of quality education, poverty, unemployment, and other social resource limitations that lead to intersubjectivity changes [16–18]. The Paulista Social Vulnerability Index (PSVI) [19] was designed to identify specific factors that lead to poverty in communities, with the aim of helping to set priorities for the health care of vulnerable populations. The PSVI considers various social determinants, such as education, health, family composition, access to public goods and services, and incorporation into the labor market. Despite the combination of economic, social, and demographic determinants of oral disease prevalence [20, 21], studies that aimed at identifying the influence of social context on perceived oral health impacts are scarce [10, 22]. Such data would contribute to the analysis of risk groups, mainly for developing public policies that positively affect adolescents’ lives.

The state of São Paulo has a Human Development Index of 0.783 (range, 0–1 from worst to best), which is the second best among Brazilian states and similar to southwestern European countries; the population of the state of São Paulo is similar to that of countries such as Poland, Ukraine, and Spain, and higher than that of Sweden, Portugal, and Belgium [23]. Among 100 Brazilian cities with the best Human Development Indices, 55 are in São Paulo [24]. Notwithstanding, this index is a broad average that does not reflect possible socioeconomic disparities across geographically similar regions. Hence, the aim of this study was to use data from the SB São Paulo 2015 survey to analyze how social vulnerability, independent socio-demographic variables, and oral health status affect perceived QoL in adolescents.

## Methods

### Study design

This analytical cross-sectional study used the PSVI and secondary data from the 2015 State Survey on Oral Health (SB São Paulo 2015) for 15–19-year-old teenagers.

### Sample size and data collection

The state survey was planned and undertaken by agents of the Brazilian Unified Health System at federal, state, and municipal levels, and by Brazilian university staff, through Ministry of Health Collaborating Centers. The aim of the survey was to assess population-wide oral health conditions across different age groups. It sampled six macroregions of São Paulo (including the State Capital, São Paulo metropolitan area, and 15 Health Departments). In the first stage, 178 cities, including the State capital of São Paulo, were designated as primary sampling units (PSUs). In the second stage, the cities were divided into 390 census tracts, including 36 tracts within São Paulo and 2 tracts within each of the remaining 177 cities.

Sampling design was carried out in conglomerates by a two-stage selection procedure with probability of selection being proportional to the population size, comprising 17,560 people in 163 cities, including 5558 participants between 15 and 19 years old. All households in the selected census tracts were contacted and eligible residents were examined and a questionnaire was applied. Those who were absent or refused to participate were excluded. The exhaustion technique with minimum sampling was applied to each PSU. More information on the sample design and the original project can be obtained in the final survey report [25].

In the SB São Paulo survey, demographic data included information on gender, age, and race/skin color, which followed the classification scheme proposed by the Brazilian Institute of Geography and Statistics (IBGE), i.e.,white, brown, black, yellow, and indigenous. Yellow, brown, and indigenous people were grouped into a single category, and the other groups were analyzed separately. Family income as reported by surveyed residents was categorized as ≤R$1500 or >R$1500.

Participants answered the nine-item OIDP questionnaire, which query how daily activities may be affected by oral conditions [7, 9]. The survey included questions aimed at investigating connections among physical, psychological, and social effects, encompassing a variety of issues ranging from eating to social interactions. Response options were: No, Yes, Do not know, and Prefer not to answer; the latter two categories were treated as missing information. The OIDP was analyzed as a dichotomous variable, considering the impact of the presence of at least one of the nine items answered affirmatively.

Dental caries and tooth loss were assessed using the Decayed, Missing, Filled-Teeth (DMF-T) index. The presence of dental caries and tooth loss were categorized as none or at least one decayed or missing tooth. Toothache was determined by the following oral morbidity question: “Did you have a toothache in the last six months?” Responses were categorized as “Yes”, “No”, and “No response”.

The Community Periodontal Index (CPI), which is recommended by the World Health Organization (WHO) for oral health surveys [26] was employed. In this way, the record considered tooth indexes (16, 11, 26, 36, 31, 46) for individuals between 5 and 19 years old. In this study, the presence of BoP and dental calculus were assessed.

Overjet was measured in millimeters and horizontally to the buccal surface of the lower incisors to the incisal edge of the most proclined incisor with CPI probes. Vertical open bite was registered as the distance, also in millimeters, between the edges of the upper and lower incisors. According to the criteria proposed by Gandini et al. [27], the distance (d’) of maxillary overjet was classified as normal (d’ = 0–3 mm), moderate (d’ > 3), or severe (d’ > 6 mm). Vertical open bite severity was classified as mild (distance ≤1 mm), moderate (1–5 mm), and severe (> 5 mm) [28]. Overjet, or anterior crossbite, was considered to be present when distances were > 1 mm.

The São Paulo Legislative institute, in partnership with Fundação Seade, used PSVI data to classify cities into six social vulnerability groups: extremely low, very low, low, medium, high, and very high social vulnerability. This classification scheme takes into consideration indicators that characterize the municipal population, such as population size, average household income, average age of household heads, number of young women responsible for the household, and number of young children (age, 0–5 years) in the total population [19]. For our study, the extremely low and very low PSVI categories were combined into our low group, the medium PSVI category was maintained, and the high and very high PSVI social vulnerability categories were combined into our high group. Data were collected from the public domain government website.

### Examiner training and calibration

Two hundred and fifty three work teams (Dentist and Oral Health Assistant) organized by the participating municipalities, participated effectively in the study. Each team was composed by one dentist and one assistant. Field teams were trained in a workshop lasting 16 h, aiming to discuss the plan of work stages, the assignments of each participant and to ensure an acceptable degree of uniformity in the procedures. Thus, only dentists performed clinical examinations (examiners) and applied the questionnaires. Oral health assistants were responsible for filling out clinical information.

Calibration was performed based on the model proposed by the WHO [26]; concordance across examiners was calculated and results were determined by team consensus. The calibration process for groups of 10 dentists had two phases: a) initial phase: presentation of clinical cases and calculation of intra-error (20 cases were presented twice with a 2 h interval) – examiners with K < 0.65 intra-examiner were eliminated from the study and K inter-examiners; b) clinical phase: clinical examinations of people in the age groups studied, and examiners with intra-examiner value less than 0.65 for caries, periodontal disease and malocclusion were eliminated from the study. If the inter-examiner value of the dentists was < 0.65, there was a new calibration round until these values were reached. The mean values were 0.82 for dental caries, 0.76 for periodontal disease and 0.74 for malocclusion.

OIDP is a validated instrument, adapted transculturally [29] and used in Brazilian population-based studies [8, 10, 11]. All teams were trained to apply the questionnaires, in order to assess the level of understanding of the questions, the time of application and operational difficulties. This questionnaire was applied to groups of 3 to 5 people per calibration group during training phase.

### Variables

**Fig. 1:**
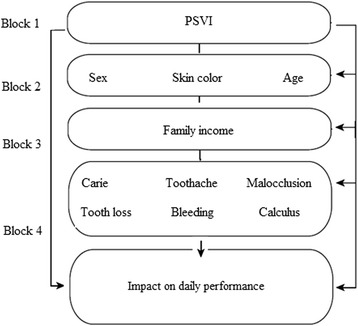
Conceptual model for the study of association among independent variables and oral health impact on the daily performance (adapted from Peres et al., 2013) [8]. *PSVI* Paulista Social Vulnerability Index

The dependent variable of the study was OIDP score. PSVI data were used to provide context together with other independent variables, such as demographic characteristics (gender, age, skin color), socioeconomic status (family income), and clinical factors (caries, tooth loss, toothache, malocclusion and periodontal changes), as shown in the conceptual model (Fig. 1).

### Data analysis

Sampling weights and conglomerates were considered in data analysis, keeping in mind the complexity of the study sampling design. Data were tabulated using Statistical Package for the Social Sciences v. 20.0 and Microsoft Excel®. A descriptive analysis was performed to obtain absolute and percentual distributions, means, medians, and standard deviations (SDs) of the variables.

A theoretical and conceptual model was used to organize the hierarchical analysis into four blocks. First, bivariate analyses were carried out between OIDP outcome and independent variables outlined in the theoretical and conceptual model. For each block, variables that presented *p* values < 0.20 were advanced to the next block, until the final model was constructed. The reference category for the binary logistic regression was “having no impact on the performance of daily activities”. A significance level of 5% was the criterion for a statistically significant effect. Odds ratios (ORs) are reported with 95% confidence intervals (CIs).

### Ethics

A survey on oral health conditions of the population in the state of São Paulo was approved by the Ethics Committee and Research with Human Beings at FOP UNICAMP in 2015 (no. 1.211.025; CAEE no. 46788215.9.0000.5418). A written informed consent was signed by every person that participated in the survey; parents or responsible guardians also signed consent forms for participants under 18 years old.

## Results

Respondents who answered “Does not know” or “did not want to answer” on an OIDP item were excluded (*N* = 156), leaving a final sample of 5402 adolescents. According to OIDP results, 57.3% of the participants did not have untreated caries, 12.7% had lost at least one tooth due to dental caries, and 27.4% reported experiencing toothache. Overjet, severe open bite, and crossbite affected 2.3, 5.7, and 1.7% of surveyed adolescents, respectively. Periodontally, 33.5% exhibited BoP and 31.4% had dental calculi (Table 1).

**Table 1:** Description of the independent variables studied in adolescents of São Paulo State (*n* = 5402)

Variable	n	% (IC95%)
PSVI		
Low	3704	71.3 (61.5; 79.5)
Medium	1521	23.8 (16.9; 32.4)
High	177	4.9 (1.4; 15.5)
Gender		
Male	2362	43.4 (40; 46.8)
Female	3040	56.6 (53.2; 60)
Skin color		
White	3213	60.2 (55.4; 64.8)
Black	401	7.6 (6.5; 8.9)
Brown	1788	32.2 (27.8; 37)
Age		
15	1616	28 (25.3; 31)
16	1016	19.1 (17.4; 20.9)
17	908	16 (14.2; 17.9)
18	863	16.8 (15.1; 18.7)
19	999	20 (17.5; 22.7)
Household income		
Up to 1.500	1909	46.1 (42.1; 50.2)
Over 1.500	2473	53.9 (49.8; 57.9)
Untreated caries lesions		
None	3149	57.3 (51.7; 62.7)
At least one	2253	42.7 (37.3; 48.3)
Tooth loss		
None	4694	87.4 (85.1; 89.3)
More than a tooth loss	708	12.6 (10.7; 14.9)
Toothache		
Yes	3996	72.7 (70; 75.2)
No	1382	27.3 (24.8; 30)
Maxillary Overjet		
Normal	3250	65.1 (60.6; 69.3)
Moderate	2013	32.6 (28.6; 36.9)
Severe	139	2.3 (1.7; 3.1)
Cross Bite		
Absence	5092	94.2 (91.0; 95.9)
Presence	310	5.8 (4.1; 8.2)
Open Bite		
Mild	4982	92.5 (89.7; 94.6)
Moderate	329	6 (4.3; 8.2)
Severe	91	1.5 (0.8; 2.8)
Presence of bleeding		
Yes	1668	32.7 (27.1; 38.8)
No	3734	67.3 (61.2; 72.9)
Presence of calculus		
Yes	1595	31.4 (25.5; 37.9)
No	3807	68.6 (62.1; 74.5)

*PSVI* Paulista social vulnerability index

**Table 2:** Sample and the quality of life distribution according to variables studied in adolescents from São Paulo (*n* = 5402)

Variable	Sample distribution	OIDP (95% CI)	
	n (%)	With impact	No impact
PSVI			
Low	3704 (71.3)	67.2 (54.8; 77.6)	73.8 (964.2; 81.6)
Medium	1521 (23.8)	25.5 (16.9; 35.9)	22.9 (15.8; 31.9)
High	177 (4.9)	7.6 (2.1; 23.9)	3.3 (1; 10.4)
Total of sample	5402 (97.19)	37.3 (33.2–41.6)	62.7 (58.4; 66.8)
Gender			
Male	2362 (43.4)	32.6 (28.9; 36.5)	46.8 (45.3; 54.3)
Female	3040 (56.6)	67.4 (63.5; 71.1)	50.2 (45.7; 54.7)
Skin color			
White	3213 (60.2)	55.5 (50.4; 60.6)	62.9 (57.3; 68.2)
Black	401 (7.6)	9.0 (7.0; 11.5)	6.8 (5.5; 8.2)
Brown	1788 (32.2)	35.4 (31.1; 39.7)	30.3 (24.8; 36.4)
Age			
15	1616 (28)	26.4 (22.8; 30.4)	29 (26.0; 32.2)
16	1016 (19.1)	17 (14.2; 20.1)	20.4 (18.4; 22.5)
17	908 (16)	17 (14.0; 20.4)	15.4 (13.4; 17.7)
18	863 (16.8)	15.2 (13.3; 17.3)	17.9 (15.4; 20.6)
19	999 (20)	24.5 (19.5; 30.3)	17.3 (15.1; 19.8)
Household income (R$)			
Up to 1.500	1909 (46.1)	50.1 (43.9; 56.2)	43.8 (38.9; 48.8)
Over 1.500	2473 (53.9)	49.9 (43.8; 56.1)	56.2 (51.2; 61.1)
Untreated caries lesions			
None	3149 (57.3)	45.2 (38.9; 51.7)	64.6 (58.5; 70.2)
At least one decayed tooth	2253 (42.7)	54.8 (48.3; 61.1)	35.4 (29.8; 41.5)
Tooth loss			
None	4694 (87.4)	82.3 (78.4; 85.6)	90.4 (88.7; 91.8)
More than one	708 (12.6)	17.7 (14.4; 21.6)	9.6 (8.2; 11.3)
Toothache			
Yes	1382 (27.3)	48.4 (44; 52.9)	14.7 (11.4; 18.8)
No	3996 (72.7)	51.6 (47.1; 56.0)	85.3 (81.2; 88.6)
Maxillary Overjet			
Normal	3250 (65.1)	60.7 (55.6; 65.5)	67.7 (62.5; 72.5)
Moderate	2013 (32.6)	36.1 (31.4; 41.0)	30.6 (26.1; 35.5)
Severe	139 (2.3)	3.2 (2.3; 4.6)	1.7 (1.1; 2.6)
Cross Bite			
Absence	5092 (94.2)	94.0 (91.2; 96)	94.3 (91.6; 96.1)
Presence	310 (5.8)	6.0 (4.0; 8.8)	5.7 (3.9; 8.4)
Open Bite			
Mild	4982 (92.5)	60.7 (55.6; 65.5)	67.7 (62.6; 72.5)
Moderate	329 (6.0)	36.1 (31.4; 41.0)	30.6 (26.1; 35.5)
Severe	91 (1.5)	3.2 (2.3; 4.6)	1.7 (1.1; 2.6)
Presence of bleeding			
Yes	1668 (32.7)	57.5 (51.6; 63.2)	26.8 (20.5; 34.4)
No	3734 (67.3)	42.5 (36.8; 48.4)	73.2 (65.6; 79.5)
Presence of calculus			
Yes	1592 (31.49)	62 (55.1; 68.5)	27.4 (20.9; 35.0)
No	3807 (68.6)	38 (31.5; 44.9)	72.6 (65.0; 79.1)

*PSVI* Paulista social vulnerability index

*OIDP* Oral impacts on daily performance

**Table 3:** Bivariate analysis and multiple regression model among independent variables and oral impact on the quality of life in adolescents from São Paulo (*n* = 5402)

	Variable	OR^a^ (IC95%)	*p* ^a^ value	OR^B^ (IC95%)	*p* ^b^ value
PSVI	Part 1				
	Low PSVI	1	< 0.01		
	Middle PSVI	1.21 (1.21; 1.21)	< 0.01		
	High PSVI	2.50 (2.49; 2.51)	< 0.01		
	Part 2				
Gender	Female	2.05 (2.04, 2.05)	< 0.01	1.78 (1.59; 2.00)	< 0.01
	Male	1	< 0.01	1	
Skin Color	White	1	< 0.01	1	< 0.01
	Black	1.51 (1.51; 1.52)	< 0.01	1.56 (1.25; 1.93)	< 0.01
	Brown	1.32 (1.32; 1.32)	< 0.01	1.66 (1.47; 1.88)	
Age group	15–19	1.09 (1.09; 109)	< 0.01	1.04 (1, 1.08)	
	Part 3				
Family income	Household income (R$)				
	Up to 1.500	1.28 (1.28; 1.29)	< 0.001	1.28 (1.28; 1.29)	< 0.001
	Over 1.500	1			1
	Part 4				
Clinical factors	Untreated caries lesions				
	None	1	< 0.01	1	
	At least one decayed tooth	2.11 (1.89; 2.36)	< 0.01	1.42 (1.25; 1.61)	< 0.01
	Tooth loss				
	None	1	< 0.01	1	
	At least one tooth loss	1.87 (1.59; 2.19)	< 0.01	1.49 (1.25; 1.78)	< 0.01
	Toothache				
	Yes	5.47 (4.80; 6.24)	< 0.01	4.87 (4.25; 5.59)	< 0.01
	No	1	< 0.01	1	
	Maxillary Overjet				
	Normal	1		1	
	Moderate	1.25 (1.11; 1.40)	< 0.01	1.23 (1.09; 1.40)	0.01
	Severe	1.80 (1.28; 2.54)	< 0.01	1.68 (1.15; 2.45)	0.006
	Cross Bite				
	Absence	1	< 0.01		
	Presence	0.94 (0.74; 1.20)	0.667		
	Open Bite				
	Mild	1		1	
	Moderate	1.34 (1.07; 1.68)	0.01	1.14 (0.89; 1.47)	0.275
	Severe	1.28 (0.84; 1.96)	0.239	1.27 (0.80; 2.03)	0.302
	Presence of bleeding				
	Yes	2.09 (1.86; 2.35)	< 0.01	1.45 (1.25; 1.68)	< 0.01
	No	1		1	
	Presence of calculus				
	Yes	2.05 (1.82; 2.31)	< 0.01	1.55 (1,34; 1,80)	< 0.01
	No	1		1	

*PSVI* Paulista social vulnerability index

*OR* ^*a*^ crude odds ratio, *OR* ^*B*^ adjusted odds ratio

*p* ^a^ value in the Wald test; *p* ^b^ value higher than 0.2 in crude or adjusted analysis

Overall, 37.3% of surveyed adolescents reported that at least one component of their daily activities was impacted by their oral health. This prevalence varied according to respondent characteristics, being higher for those with oral diseases, as well as for females, persons who considered their skin color to be black or brown, 19-year-olds, and those with family income ≤ R$1500 per month (Table 2).

Results of our bivariate analysis and the hierarchical analysis adjustments are described in Table 3. In the crude analysis, all variables, except for crossbite, were associated with a high OIDP score and were included in the adjusted model.

The final model of the multiple logistic regression analysis was adjusted in accordance with each block. The only independent variable that lost its significance status after block-one adjustment was age (*p* value > 0.2). In block two, gender remained a significant factor, with females reporting a greater impact on OIDP than males (OR 1.78, 95% CI = 1.59–2.00; *p* < 0.01), as did skin color, with the reported impact higher among adolescents with brown skin (OR 1.66, 95% CI = 1.47–1.88; *p* < 0.01). In block three, family income was found to be a significant factor, with the reported impact of oral conditions on QoL higher among those with a monthly family income up to R$1500 (OR 1.28, 95% CI = 1.28–1.29; *p* < 0.01). In different oral disease states allocated in block four, several conditions were found to affect self-perceived QoL of adolescents, including at least one untreated decayed tooth (OR 1.42, 95% CI = 1.25; 1–61; *p* < 0.01), loss of one or more teeth (OR 1.49 95% CI = 1.25–1.78]; *p* < 0.01), toothache (OR 4.87, 95% CI = 4.25–5.59; *p* < 0.01), BoP (OR 1.45, 95% CI = 1.25–1.68; *p* < 0.01), presence of dental calculus (OR 1.55, 95% CI = 1.34–1; *p* < 0.01), and severe maxillary overjet (OR 1.68, 95%CI = 1.15–2.45; *p* < 0.006) (Table 3).

## Discussion

In the present study sample, 37.7% of surveyed 15–19-year-olds reported that their oral health had at least some impact on their daily activities, a percentage similar to 39.4% obtained in the SB Brazil 2010 survey [8]. Greater impact, as indexed by the OIDP score, was associated with females, brown skin color, a low household income, and the presence of oral abnormalities.

Our finding of females reporting a higher impact of dental problems on quality of life is consistent with several previous studies [8, 10, 13]. This gender effect may be caused by the fact that girls are more self-critical of their dental appearance and have a lower self-esteem than boys [30]. These characteristics may make girls particularly sensitive to malocclusion and untreated caries [11, 12].

Several research groups have discussed the effects of malocclusion on QoL in adolescents [11, 14, 31], with some attempting to elucidate which kinds of dental positioning problems are most likely to have a negative effect on self-perceived QoL in different age groups [30–33]. Crowded teeth affecting smiling had a negative impact as a malocclusion condition [30]. Among 18-year-olds, a severe maxillary overjet (> 5 mm) was reported to be associated with a 3.7 times greater likelihood of seeking intervention related to emotional state [32]. This prior result is consistent with our finding of maxillary overjet remaining a significant factor in the final model of our regression analysis. It is worth mentioning that the SB São Paulo survey criteria for malocclusion included specifically maxillary overjet, cross bite, and open bite, which limits the assessment of other aspects of dentition positioning and esthetics.

The presence of caries, toothache, and tooth loss affected self-reported QoL and is consistent with prior studies [8, 32]. These conditions often lead to discomfort, which may affect social interactions, limit masticatory function, and impair one’s ability to concentrate [15]. Consistent with the present results, Leo et al. [34] found that untreated caries had a negative impact on QoL and social relationships of adolescents in a vulnerable community in the countryside of São Paulo.

Our findings that BoP and the presence of dental calculi were factors associated with impacted daily activities of young people corroborate the findings of Vazquez et al. [10]. The presence of calculi and gingivitis have been reported previously to have consequential psychosocially impacts on whether a person can smile without being embarrassed about his or her teeth and making people not to brush their teeth due to the fear of bleeding gums [15]. A previous research on school-aged children has also linked dental plaque and oral health to a worse QoL [35].

The hypothesis that social vulnerability is associated with oral health impacts on QoL was based on prior evidence indicating that geographical and socio-demographic disparities affect the incidence of oral diseases [21]. Low human development index for the black population has been associated with the prevalence of dental caries, tooth loss, the need for prosthesis, and edentulism [20, 21]. In the present study, PSVI, an assessment of context, was not a significant factor in determining whether adolescents’ daily activities were affected by their oral health. Similarly, a prior social context study on 1172 15–19-year-olds in São Paulo did not find an association between OIDP score and social exclusion [10].

In the Bourdie system, perception is a component of *habitus*, defined as the socialized norms or tendencies that guide behavior and thinking [36]. Accordingly, perceived QoL may be a product of interactions between the individual and collective biographical experiences, and may transcend economic influences. Regardless, ethnic and socioeconomic issues remain factors in perception. For example, oral health impact on QoL of adolescents was found in one study to be 1.44 times and 2.41 times greater in individuals with brown skin or low income, respectively [8]. This disparity may be related to a lack of access to goods and basic services, in addition to exposure to various risk and behavioral factors, which are dissociable from racial identity per se.

Concepts of oral disease that focus solely on functional and morphological issues are based on a biochemical view of medicine that does not consider the individual holistically [16]. However, oral health problems have effects that go beyond clinical factors. Hence, for us, dentistry should consider aspects of the patient beyond pathology, several of which can be probe with the OIDP.

Understanding adolescents’ perceptions of their oral health conditions can contribute to the development of behavior modification strategies aligned with current health regulations [6]. Habits that can lead to serious dental and gingival changes in adolescents include carelessness with oral hygiene and a cariogenic diet [37, 38]. Young people often do not realize that they need dental treatment until their oral health conditions affect their daily activities. Generally, this segment of the population seeks dental care when they have a problem such as a toothache, rather than seeking preventive care [34]. Therefore, in addition to expanding the provision of dental services, it is important to develop health-promoting educational strategies aimed at teaching teenagers about their needs and strengthening their oral health care proactivity.

When interpreting the effects of this study, it is important to consider its limitations. Because of the cross-sectional design, causal relationships cannot be established, making it difficult to determine whether factors precede or follow associated outcomes. **Another possible limitation of the study was that the data were collected by the dentist and not by a trained interviewer. Therefore, having the clinical examiner also administered the questionnaire may be indicative of bias in the information collected.** Conversely, this study has notable strengths, including the use of data from a broad-sample survey representative of some 533 million adolescents [39], which makes data relevant for other Brazilian states and other regions and countries with similar characteristics.

## Conclusion

The present results indicate that the impact of oral health on adolescents self-perceived QoL is related more to clinical pathological factors than social vulnerability.

The degree of vulnerability of the municipalities in the state of São Paulo did not influence the impact of oral health on daily activities of adolescents. However, the impact of oral health on adolescents’ daily activities was found to be sensitive to socio-demographic determinants (i.e., gender, skin color, and income) and oral clinical variables (i.e., dental caries, tooth loss, periodontal conditions, and malocclusion). These results may contribute to the planning of strategies to improve oral health actions.

## Abbreviations

CPI: Community Periodontal Index
DMF-T: Decayed, Missing, Filled index
IBGE: Brazilian Institute of Geography and Statistics
ICF: Informed Consent Term
OIDP: Oral Impacts on Daily Perfomance
PSU: Primary sampling unit
PSVI: Paulista Social Vulnerability Index
SD: Standard deviation
WHO: World Health Organization

## References

[1] Brazil. Ministry of Health. Secretariat of Health Care. Department of Strategic Programmatic Actions. Adolescent health: skills and abilities / Ministry of Health, Health Care Secretariat, Department of Strategic Programmatic Actions. - Brasília: Publisher of the Ministry of Health. 2008.

[2] Veiga NJ, Pereira CM, Ferreira PC, Correia IJ (2014). Oral health behaviors in a sample of Portuguese adolescents: an educational issue. Health Promot Perspect.

[3] Alves FNM, Andrade CLT, Vettore MV (2015). Planning oral health care using the sociodental approach and the index of family living conditions: a cross-sectional study in Brazilian adolescentes. BMC Res Notes.

[4] Brazil. Ministry of Health. Secretariat of health care. SB Brazil project 2003: oral health conditions of the Brazilian population 2002–2003. Main results. Brasília: Ministry of Health. 2004.

[5] Brazil. Ministry of Health. Secretariat of Health Surveillance. Secretariat of Health Care. National Coordination of Oral Health. SB2010. National Oral Health Survey. Main results. Brasília: MS. 2011.

[6] Barbosa TS, Mialhe FL, Castilho ARF, Gavião MBD (2010). Quality of life and oral health in children and adolescents: conceptual and methodological aspects. Physis.

[7] Adulyanon S, Vourapukjaru J, Sheiham A (1996). Oral impacts affecting daily performance in a low dental disease Thai population. Community Dent Oral Epidemiol.

[8] Peres KG, Cascaes AM, Leão ATT, Côrtes MIS, Vettore MV (2013). Socio-demographic and clinical aspects of oral health-related quality of life in adolescents. Rev Saúde Pública.

[9] Hongxing L, List T, Nilsson I, Johansson A, Astrøm AN (2014). Validity and reliability of OIDP and OHIP-14: a survey of Chinese high school students. BMC Oral Health.

[10] Vazquez FL, Cortellazzi KL, Kaieda AK, Guerra LM, Ambrosano GM, Tagliaferro EP, Mialhe FL, Meneghim MC, Pereira AC (2015). Quality of life and socio-dental impact among underprivileged Brazilian adolescents. Qual Life.

[11] Bernabé E, Tsakos G, Messiah OC, Sheiham A (2008). Impacts on daily performances attributed to malocclusions using the condition-specific feature of the oral impacts on daily performances index. Angle Orthod.

[12] Choi SH, Kim BI, Cha JY, Hwang CJ (2015). Impact of malocclusion and common oral diseases on oral health-related quality of life in young adults. Am J Orthod Dentofac Orthop.

[13] Krisdapong S, Prasertsom P, Rattanarangsima K, Sheiham A (2014). Associations between perceived needs for dental treatment, oral health-related quality of life and oral diseases in school-aged Thai children. Community Dent Oral Epidemiol.

[14] Dimberg L, Arnrup K, Bondemark L (2015). The impact of malocclusion on the quality of life among children and adolescents: a systematic review of quantitative studies. Eur J Orthod.

[15] Krisdapong S, Prasertsom P, Rattanarangsima K, Sheiham A (2012). Relationships between oral diseases and impacts on Thai schoolchildren ' s quality of life: evidence from the Thai national oral health survey of 12-and 15-year-olds. Community Dent Oral Epidemiol.

[16] Czeresnia D, Freitas CM (2009). Health promotion: concepts, reflections, trends. 2nd.

[17] Fonseca FF, Sena RKR, Santos RLA, Dias OV, Veloso DO, Melo SC (2013). The vulnerabilities in childhood and adolescence and the Brazilian public policy intervention. Rev paul pediatr.

[18] Ayres JRCM, França Júnior I, Calazans GJ, Saletti Filho, HC. The concept of vulnerability and health practices: new perspectives and challenges In: Czeresnia D, Freitas CM. (Orgs.) Health promotion: concepts, reflections, trends. Rio de Janeiro: Fiocruz, 2003. p.117-139.

[19] Legislative Assembly of the State of São Paulo. Paulista Social Vulnerability Index, version 2010. http://indices-ilp.al.sp.gov.br/view/index.php?prodCod=2. 2016; Accessed 04 June 2016

[20] Guiotoku SK, Moysés ST, Moysés SJ, França BHS, Bisinelli JC (2012). Racial iniquities in oral health in Brazil. Rev. Panam Salud Pública..

[21] Frias AC, Antunes JLF, Junqueira SR, Narvai PC (2007). Individual and contextual determinants of the prevalence of untreated dental caries in Brazil. Rev Panam Salud Pública.

[22] Martins MT, Sardenber GF, Vale MP, Paiva SM, Pordeus IA (2015). Dental caries and social factors: impact on quality of life in Brazilian children. Braz oral res.

[23] United Nations Development Program - UNDP (2014). Human Development Report 2014 - Sustaining human progress: Reducing vulnerabilities and enhancing resilience. http://hdr.undp.org/sites/default/files/hdr2014_pt_web.pdf. Accessed 30 Aug 2016.

[24] United Nations Development Program - UNDP (2013). Atlas of Human Development in Brazill. http://www.atlasbrasil.org.br/2013/pt/perfil_uf/sao-paulo. Accessed 07 Aug 2016.

[25] Pereira AC, Vladen V, Frias, AC. Oral health state survey: final report. - Waters of St. Peter, 2016.

[26] World Health Organization (1997). Oral heath surveys: basic methods.

[27] Gandini MREAS, Pinto AS, Gandini MJCR, Mendes AJ (2000). Study of dental occlusion of schoolchildren from the city of Araraquara, in the mixed denture phase, inter-arches, anterior region (overjet and overbite). Ortodontia.

[28] Dawson PE (1974). Solving anterior open bite problems. In: evaluation, diagnosis and treatment of occlusal problems.

[29] Goes PSA (2001). The prevalence and impact of dental pain in Brazilian schoolchildren and their families [doctoral dissertation].

[30] Marques LS, Ramos-Jorge ML, Paiva SM, Pordeus IA (2006). Malocclusion: esthetic impact and quality of life among Brazilian schoolchildren. Am J Orthod Dentofac Orthop.

[31] Chen M, Cz F, Liu X, Li ZM, Cai B, Wang DW (2015). Impact of malocclusion on oral health-related quality of life in young adults. Angle Orthod.

[32] Traebert ES, Peres MA (2007). Do malocclusions affect the individual's oral health-related quality of life?. Oral Health Prev Dent.

[33] Claudino D, Traebert J (2013). Malocclusion, dental aesthetic self-perception and quality of life in the 18 to 21 year-old population: a cross section study. BMC Oral Health.

[34] Leão MM, Garbin CAS, Moimaz SAS, Rovida TAS (2015). Oral health and quality of life: an epidemiological survey of adolescents from settlement in Pontal do Paranapanema/SP. Brazil Ciênc saúde coletiva.

[35] Schuch HS, Costa FS, Torriani DD, Demarco FF, Goettems ML (2015). Oral health-related quality of life of schoolchildren: impact of clinical and psychosocial variables. Int J Paediatr Dent.

[36] Bourdie P (1996). Razões práticas: sobre a teoria da ação.

[37] Peres MA, Sheiham A, Liu P, Demarco FF, Silva AE, Assunção MC, Menezes AM, Barros FC, Peres KG (2016). Sugar consumption and changes in dental caries from childhood to adolescence. J Dent Res.

[38] Hall-Scullin E, Goldthorpe J, Milsom K, Tickle M (2015). A qualitative study of the views of adolescents on their caries risk and prevention behaviours. BMC Oral Health.

[39] Brazilian Institute of Geography and Statistics. Population count. http://www.ibge.gov.br/home/estatistica/populacao/contagem2007/default.shtm. Accessed 12 Sept 2016.

